# Supplementary material for: Coevolutionary Immune System Dynamics Driving Pathogen Speciation
Source: PLoS One. 2014 Jul 23;9(7):e102821. doi: 10.1371/journal.pone.0102821 (PMC4108359; doi:10.1371/journal.pone.0102821)
Supplement: Appendix S1 — Estimation and discussion of parameter values. (PDF) [file pone.0102821.s001.pdf]

Appendix S1. Estimation and discussion of parameter values.

This Appendix provides biological details and discussion on parameter choices used in this model. To obtain insight into the co-evolutionary dynamics of rapidly mutating pathogens and the adaptive immune response, we choose biologically relevant values for these parameters (given in Table 1 in the main text), drawing from previously measured and modeled characteristics of T-cells and well-studied viruses.

The parameter values shown in Table S1 are adopted or slightly modified from a previously published shape space model of T-cell dynamics [1] or a related model of thymic T-cell influx during chronic infection [2], in order to reproduce the basic acute infection immune system dynamics seen in these models. The rate of pathogen growth λ is consistent with [1], and similar to previously measured values of the initial expansion rate of HIV-1 [3, 4]. The rate of pathogen killing by the immune response has been estimated to be between 0.1 and 0.5 day^{-1} near the peak of viremia in HIV and SIV infections [5, 6]. The concentration of virus-specific T-cells responding to the primary infection near this peak can be roughly approximated as $10^4\ \mu\text{l}^{-1}$, based on measurements of the CD8 T-cell response to an infection of lymphocytic choriomeningitis virus (LCMV) in the mouse spleen [7, 8]). Given these estimates, a value on the order of $10^{-5}\ \mu\text{l} \cdot \text{day}^{-1}$, as used in [1] and [2], is reasonable for a pathogen killing rate per day, per concentration unit of T-cells in the adaptive response.

The T-cell replication and decay rates used here (denoted σ and δ, respectively) are consistent with those estimated from measurements of the immune response to LCMV in mice (replication rates between 1 and 3 day^{-1}, decay rates between 0.3 and 0.5 day^{-1}) [8, 9]. An approximate stimulation coecient κ is given by early-stage HIV data in [10], in which the significant T-cell response is observed to begin when viremia is on the order of $10^5\ \mu\text{l}^{-1}$.

From a previous shape space modeling paper [1], we adopt an initial naïve T-cell concentration L_0 of 3 μl^{-1} for each shape space site (T-cell clone). This is a biologically reasonable value with typical overall naïve T-cell concentrations. We can then estimate Γ, the naïve cell inux at a single shape space site (T-cell clone), from the initial equilibrium condition in the absence of pathogen, given in Eq. 10 in the main text: setting $L0 = \Gamma/\delta$ gives an approximate value of $\Gamma = 1\ (\mu\text{l} \cdot \text{site} \cdot \text{day})^{-1}$. We chose a value of $\phi = 10^{10}\ \mu\text{l}^{-1}$ for the pathogen carrying capacity in order to give acute infection dynamics comparable to those in [10] and [1].

Table S1: Parameters adopted from previously published models [1, 2], and consistent with several measurement and estimation studies on immune system and virus properties (see text).

Parameter	Sym.	Value
pathogen growth	λ	3 day^{-1}
pathogen killing	β	$10^{-5}\ \mu\text{l} \cdot \text{day}^{-1}$
naïve cell influx	Γ	$1\ (\mu\text{l} \cdot \text{site} \cdot \text{day})^{-1}$
T-cell replication	σ	3 day^{-1}
T-cell decay	δ	0.33 day^{-1}
stimulation coefficient	κ	$10^5\ \mu\text{l}^{-1}$

We express mutation in terms of the overall mutation rate μ of the pathogens, in the commonly used units of substitutions per DNA base per replication cycle. Each epitope has on the order of

10 amino acids, for an estimate of about 30 base pairs; thus, under the assumption that all base pairs in the pathogen genome mutate independently at identical rates, we approximate the expected number of mutations per cycle for a given epitope $\vec{x}$ as

$$\mu'(\vec{x}) \approx 30\mu. \tag{1}$$

Consequently, if every member of a population of P identical epitopes replicates once, we will have $P \times 30\mu$ mutants. In terms of our model, then, $\mu'(\vec{x})$ can be interpreted as the fraction of pathogens at location $\vec{x}$ that differentiate to locations $\vec{x}' \neq \vec{x}$, determined by the normalized mutation kernel Q and the simulation space size n:

$$\mu'(\vec{x}) = \sum_{\vec{x}' \neq \vec{x}} Q(\vec{x}, \vec{x}') = \chi \left(\frac{2}{\pi}\right)^{\frac{1}{2}} \sum_{\vec{x}' \neq \vec{x}} |\vec{x} - \vec{x}'|^{-2}. \tag{2}$$

Here, the sum is performed over all $n-1$ sites $\vec{x}'$ that are not equal to $\vec{x}$. For our chosen kernel Q, as given in Eq. 7 in the main text of the paper, and $n = 400$, μ' is nearly constant across the simulation space (i.e., $< \mu'(\vec{x}) > \approx \mu'(\vec{x}),\ \forall\ \vec{x}$), so we set its average value equal to our approximation in Eq. 1:

$$< \mu'(\vec{x}) > = \frac{\chi}{n} \left(\frac{2}{\pi}\right)^{\frac{1}{2}} \sum_{\vec{x}} \sum_{\vec{x}' \neq \vec{x}} |\vec{x} - \vec{x}'|^{-2} \approx 30\mu. \tag{3}$$

Thus, having chosen Q and n, the value of the parameter χ is uniquely determined from μ. In this study, we use the standard value $\mu = 2.2 \times 10^{-5}$ substitutions per base pair per cycle, similar to commonly measured values for the rapidly mutating virus HIV-1 [11], and study the effects of variation between 1×10^{-5} and 5×10^{-5}. The binding specificity parameter b gives the width of the Gaussian affinity curve in shape space, setting the specificity of the naïve T-cell response. We choose b to correspond to typical values of the precursor frequency, or the probability of two different naïve T-cells responding to the same epitope (about $1/10^5$ [12, 13]). Starting from the uniformly distributed initial condition in which $L(\vec{y}) = \Gamma/\delta\ \forall\ \vec{y}$ (Eq. 10 in the main text of the paper), the number of naïve T-cells under the affinity curve centered at some $\vec{y_0}$, and thus cross-reactive with the response to a pathogen maximally complementary to $\vec{y_0}$, is given in one dimension by

$$\frac{\Gamma}{\delta} \int \gamma(\vec{y}, \vec{y_0})\ d\vec{y} = \frac{\Gamma}{\delta} \int \exp\left[-\frac{|\vec{y} - \vec{y_0}|^2}{2b^2}\right]\ d\vec{y} = \frac{b\Gamma\sqrt{2\pi}}{\delta}. \tag{4}$$

Although we only simulate a small portion of the full shape space for computational efficiency, the total number of T-cells in our system is approximated by the initial naïve density times the total number of clones in the human immune system, with reasonable values between $10^6 - 10^7$ [12]. To estimate b, we can then set the fraction of cross-reactive naïve T-cells equal to the precursor frequency:

$$b\sqrt{2\pi}/(5 \times 10^6) = 10^{-5} \quad \longrightarrow \quad b \approx 20. \tag{5}$$

In this paper, we investigate values of b corresponding to the above range of total naïve T-cell clones in the system.

We investigated the system under several values of ξ, which controls the strength of the damping homeostatic pressure. We chose values small enough that $\xi << \sigma R/|L_{tot} - R|$ and $\xi << \delta R/|L_{tot} - R|$, so that the homeostatic pressure would not significantly alter the expected chronic infection

steady-state levels. In the main text of the paper we present results obtained at two different values of ξ. The higher value (Fig. 5 in main text) shows much stronger damping of total population density oscillations in acute infection, and converges to approximate chronic equilibrium quickly. Infections at the lower value of ξ (Fig. 4 in main text) take longer to converge to a chronic infection, branch, and eventually escape control. The branching and escape behaviors are observed consistently during chronic infections across values of ξ, and appear to be driven by the same mechanisms, although the timescale on which they occur changes, as well as the precise values of other parameters needed to generate a chronic infection.

In order to ensure that our choice to simulate only a limited region of the total shape space of naïve T-cell clones does not substantially affect our results, we investigated the behavior of several infections in a shape space with a substantially larger T-cell population (between $10^6 - 10^7$ naïve clones). We found that extending the shape space in this way is effectively the same as lowering the parameter ξ: as discussed in the main text, the timescales of convergence to chronic infection, branching, and eventual immune escape are lengthened in a large shape space, while the overall effectiveness of the immune system is enhanced. However, the mechanisms leading to chronic infection, branching, and eventual pathogen escape are similar, though occurring on different timescales. In the main text and figures, we thus focus on presenting results from a small portion of shape space for computational efficiency and more effective visualization.

References

[1] Stromberg SP, Carlson JM (2013) Diversity of T-cell responses. Physical Biology 10: 025002.

[2] Stromberg SP, Antia R (2012) On the role of CD8 T cells in the control of persistent infections. Biophysical Journal 103: 1802–1810.

[3] Little SJ, McLean AR, Spina CA, Richman DD, Havlir DV (1999) Viral dynamics of acute HIV-1 infection. The Journal of experimental medicine 190: 841–850.

[4] Ribeiro RM, Qin L, Chavez LL, Li D, Self SG, et al. (2010) Estimation of the initial viral growth rate and basic reproductive number during acute HIV-1 infection. Journal of Virology 84: 6096–6102.

[5] Ganusov VV, De Boer RJ (2006) Estimating costs and benefits of CTL escape mutations in SIV/HIV infection. PLoS Computational Biology 2: e24.

[6] Goonetilleke N, Liu MK, Salazar-Gonzalez JF, Ferrari G, Giorgi E, et al. (2009) The first T cell response to transmitted/founder virus contributes to the control of acute viremia in HIV-1 infection. The Journal of Experimental Medicine 206: 1253–1272.

[7] Murali-Krishna K, Altman JD, Suresh M, Sourdive DJ, Zajac AJ, et al. (1998) Counting antigen-specific CD8 T cells: a reevaluation of bystander activation during viral infection. Immunity 8: 177–187.

[8] De Boer RJ, Oprea M, Antia R, Murali-Krishna K, Ahmed R, et al. (2001) Recruitment times, proliferation, and apoptosis rates during the CD8+ T-cell response to lymphocytic choriomeningitis virus. Journal of Virology 75: 10663–10669.

[9] De Boer RJ, Homann D, Perelson AS (2003) Different dynamics of CD4+ and CD8+ T cell responses during and after acute lymphocytic choriomeningitis virus infection. The Journal of Immunology 171: 3928–3935.

[10] Davenport MP, Ribeiro RM, Perelson AS (2004) Kinetics of virus-specific CD8+ T cells and the control of human immunodeficiency virus infection. Journal of Virology 78: 10096–10103.

[11] Sanjuán R, Nebot MR, Chirico N, Mansky LM, Belshaw R (2010) Viral mutation rates. Journal of Virology 84: 9733–9748.

[12] Perelson AS, Weisbuch G (1997) Immunology for physicists. Reviews of Modern Physics 69: 1219.

[13] Blattman JN, Antia R, Sourdive DJ, Wang X, Kaech SM, et al. (2002) Estimating the precursor frequency of naive antigen-specific CD8 T cells. The Journal of Experimental Medicine 195: 657–664.
